# Development of a 3D seed morphological tool for grapevine variety identification, and its comparison with SSR analysis

**DOI:** 10.1038/s41598-018-24738-9

**Published:** 2018-04-25

**Authors:** Avshalom Karasik, Oshrit Rahimi, Michal David, Ehud Weiss, Elyashiv Drori

**Affiliations:** 1 The National Laboratory for Digital Documentation and Research in Archaeology, Israel Antiquities Authority, Jerusalem, Israel; 2 Department of Agriculture and Oenology, Eastern regional R&D center, Ariel University, Ariel, Israel; 3 Martin (Szusz) Department of Land of Israel Studies and Archaeology, Bar-Ilan University, Ramat-Gan, Israel; 4 Department of Chemical Engineering, Biotechnology and Materials, Ariel University, Ariel, 40700 Israel

## Abstract

Grapevine (*Vitis vinifera* L.) is one of the classical fruits of the Old World. Among the thousands of domesticated grapevine varieties and variable wild *sylvestris* populations, the range of variation in pip morphology is very wide. In this study we scanned representative samples of grape pip populations, in an attempt to probe the possibility of using the 3D tool for grape variety identification. The scanning was followed by mathematical and statistical analysis using innovative algorithms from the field of computer sciences. Using selected Fourier coefficients, a very clear separation was obtained between most of the varieties, with only very few overlaps. These results show that this method enables the separation between different *Vitis vinifera* varieties. Interestingly, when using the 3D approach to analyze couples of varieties, considered synonyms by the standard 22 SSR analysis approach, we found that the varieties in two of the considered synonym couples were clearly separated by the morphological analysis. This work, therefore, suggests a new systematic tool for high resolution variety discrimination.

## Introduction

Grapevine (*Vitis vinifera* L.) is one of the classical fruits of the Old World; it comprises an important part of the oldest group of fruit trees around which horticulture evolved in the Mediterranean basin^1^. *Vitis* pips (seeds) are rounded or ovoid, mostly gradually tapering into a beak, ending into the micropile. The dorsal side is swollen with a deep furrow and enlarged in the center, the chalaza. The ventral side has two fossettes separated by the raphe, a prominent ridge^2–4^. Based on the fact they are highly polymorphic, grape pips have been used as a basis for taxonomy within the genus *Vitis*, specifically for the grapevine^4^. Wild *sylvestris* grapes may be recognized by more numerous (3–4) pips per berry, and by somewhat more globular pips, generally with beaks constricted at the attachment to the main body of the pip. Among the thousands of domesticated grape vine varieties and among the variable wild *sylvestris* populations, the range of variation in pip morphology is very wide. Furthermore, the variation in the domesticated assemblage and in the wild forms overlaps considerably. For this reason pip morphology was not considered traditionally as a fully safe diagnostic trait for distinguishing between wild and domesticated forms^1^.

However, several attempts were made recently to solve the issue of grapevine pips as a diagnostic tool, using present-day image analysis techniques. Spearheaded by Terral^5–9^, these studies applied geometrical analysis (elliptic Fourier transform method) to analyze the 2D outlines of grapevine pips. The investigation of variation in pip morphology aims to provide accurate criteria for the quantification of phenotypic diversity of various grape varieties, wild as well as modern cultivars. In addition, this approach was used to understand changes related to the domestication process and to investigate archaeological finds. Of specific importance is Terral *et al*., (2010) paper, which demonstrate the applicability of geometrical analysis approach to grape pips. In this paper, they created a morphological key for pips of some fifty French grapevine varieties and demonstrated significant correlation between pip morphology and taxonomic relationship. Similar method was recently used by Sabato *et al*.^10^ to classify and compare archeological seeds of melon (*Cucumis melo* L.) against a modern collection of melon seeds. They extract 18 morphological parameters from 2D images of the seeds and used them in their classification^10^.

In the last decade, 3D scanning technology has advanced dramatically. In addition to its significant role in industry, modern academic studies harnessed this technology to explore and investigate new questions that were never accessible without the 3D information. For instance, in the analysis of flint tools in archaeology, the center of gravity, which cannot be measured without accurate 3D models, was used as a parameter for the flint tools analysis^11^. Many researches have shown the potential of the combination of a very accurate measuring technique in 3D, together with mathematical and statistical methods as well as innovative algorithms from the field of computer sciences^12–14^. Generally we can say that the great advantage of using 3D for morphological studies is that it brings systematic and objective methods into the field of shape descriptor which very often can be vague and subjective. In this paper we describe our efforts at developing a 3D tool for grape variety identification. The work was done by 3D scanning of representative samples of fresh grape pip populations, including Israeli indigenous *sativa* and *sylvestris* populations collected in our previous work^15,16^, as well as international varieties.

Nevertheless, as genetic identification is the main approach used today for variety identification, we used the genetic approach to verify our 3D identification results. As the grapevine is a major economic crop, its genome was one of the first amongst tree species to be fully sequenced and assembled. Moreover, grapevine genome is one of the smallest among crop plants, ca. 500 Mb^17^, which makes genomic research for this plant more approachable. Large sets of grapevine genes were annotated in current databases available to the research community. Nevertheless, Simple Sequence Repeat (SSR) analysis is still the main genetic method for the identification of grapevine varieties^18,19^. In this paper, we used this tool as a reference, and compared the results achieved with both methods. Recently, Bacilieri *et al*.^20^ proved the applicability of such approaches to well-preserved archaeological find, and concluded that the combined use of Single Nucleotide Polymorphisms (SNP) markers and morphogeometry is promising for deciphering the intricate history of grapevine domestication.

## Results

**Figure 1:**
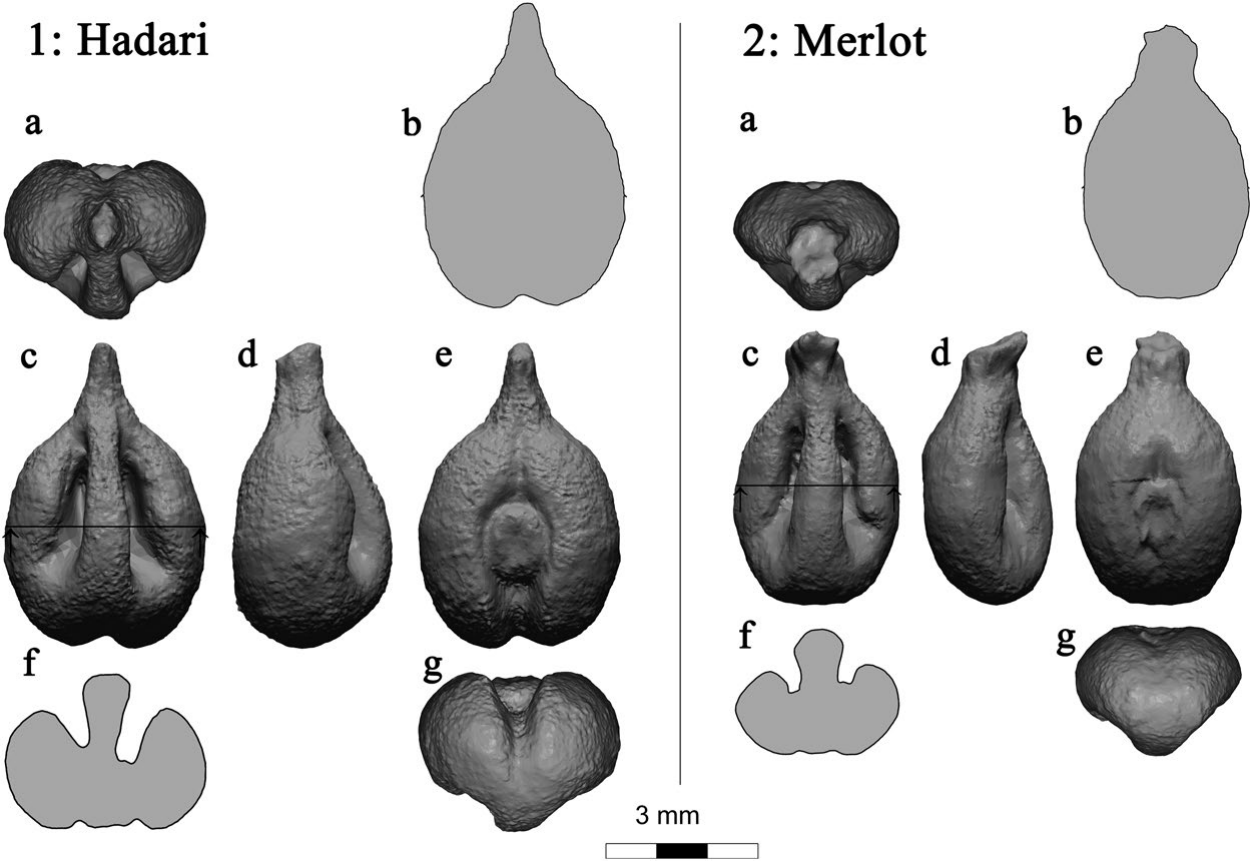
3D rendered views (**a**,**c**,**d**,**e**,**g**) and cross-sections (**b**,**f**) of grape pips from two varieties: 1: Hadari (indigenous Israeli variety - left), 2: Merlot (international variety - right).

**Figure 2:**
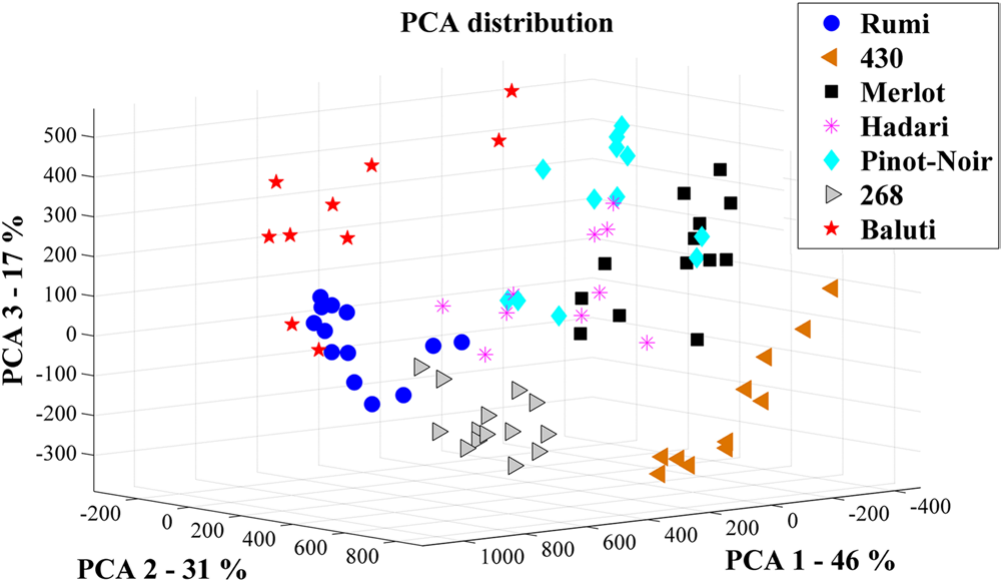
PCA distribution of the assemblage of seven grape varieties, using the optimal set of weights. It shows a very clear separation between six varieties, with very few overlaps, and the variety Hadari is relatively spread and overlaps with other groups.

**Figure 3:**
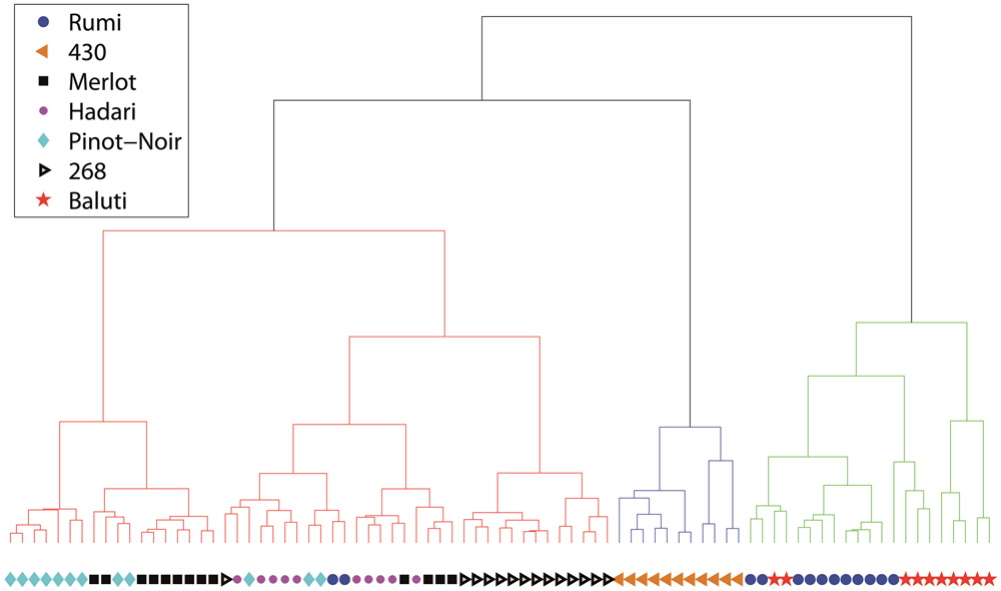
Cluster tree for the assemblage of seed morphology resemblance of the seven grape types, when the optimal set of weights of the Fourier parameters were used.

In order to show the potential of our classification method and its capability to differentiate between various grapevine varieties, based only on their seed’s morphology, we have first selected seven types of grape seeds. Two varieties, Merlot (N = 13) and Pinot-Noir (N = 12) are well-known international varieties; three varieties, Rumi (N = 13), Baluti (N = 10), and Hadari (N = 10) are local traditional Israeli varieties; and two varieties, ‘430’ (N = 11) and ‘268’ (N = 14) are *Vitis vinifera* ssp*. sylvestris* lines which were found in our survey as was described in the introduction. We scanned each seed using a high-resolution 3D scanner. Two examples of scanned seeds with five perpendicular views and cross-sections can be seen in Fig. 1. The 3D information was characterized using several planar curves which represent key features of the 3D object. Two representative planar curves are shown in Fig. 1 views 1-f and 2-f. Another significant planar curve that we used is the silhouette of the seeds extracted from their optimal positioning, as can be seen in view b of each example. For further details on the scans and shape characteristics please refer to the *materials and methods* section. Using the selected Fourier coefficients, a very clear separation was obtained between most of the varieties, with only very few overlaps. A Principal Components Analysis (PCA) plot (Fig. 2) and the cluster tree (Fig. 3) based on this analysis present the good separation obtained. At the Supplementary Information we present an analysis with a different set of weights in which the separation is less optimal (see Supplementary Fig. S1).

Analysis of the cluster tree produced from the morphological data using the optimal set of Fourier coefficients (Fig. 3) shows that the local Israeli varieties Baluti and Rumi are nicely separated and cluster together, ‘430’ *sylvestris* line stands alone, and most of the seeds of the varieties Pinot-Noir and Merlot are nicely separated and cluster together. In another sub cluster the ‘268’ *sylvestris* line stands alone. A less distinct separation was achieved for the Hadari variety, which stands in a mixture with a few Rumi, Merlot and Pinot-Noir seeds, and one ‘268’ seed.

**Figure 4:**
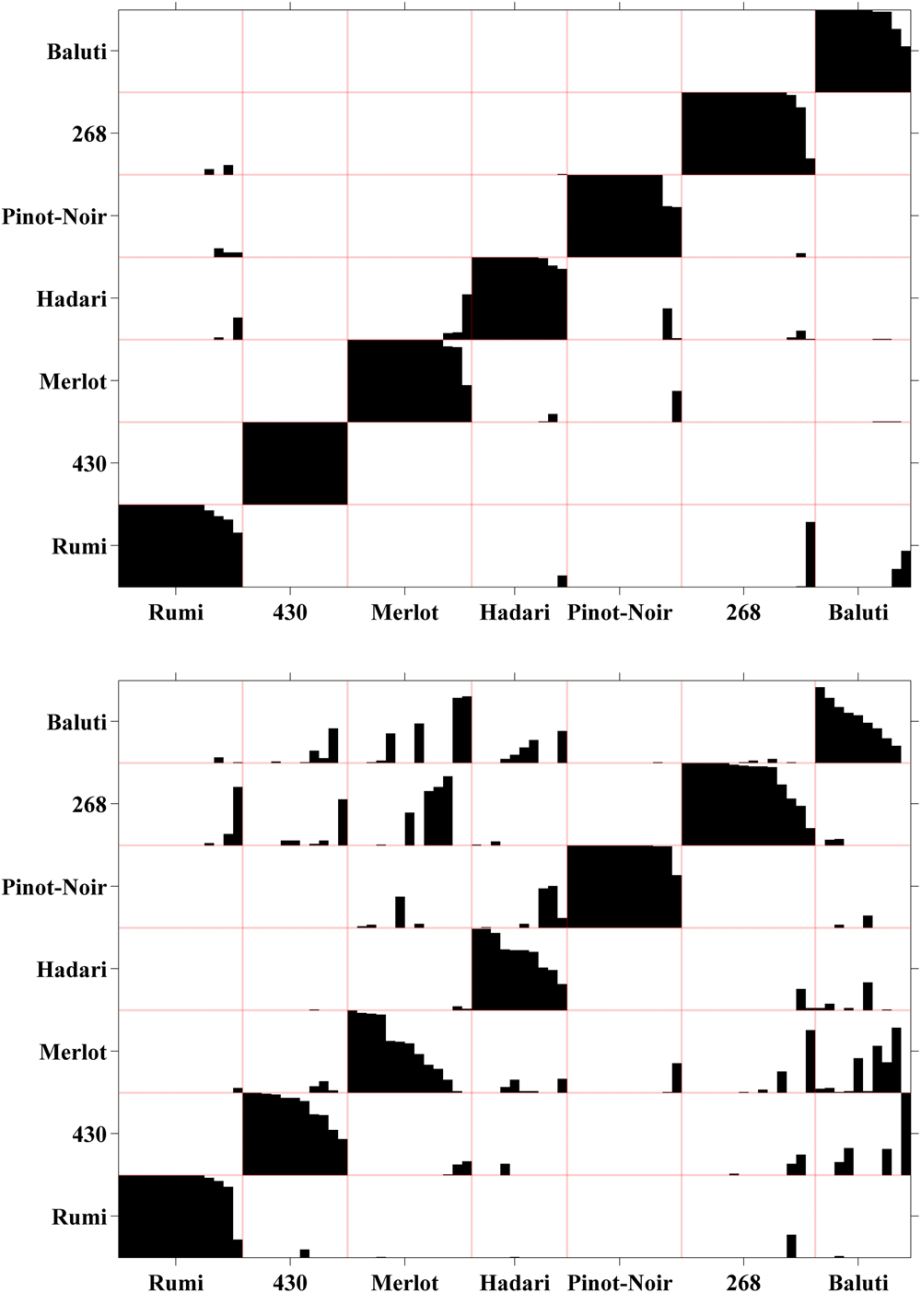
Accumulative distribution of Discriminant Analysis classification after running 1000 blind-tests. At each run, half of the assemblage was randomly selected as the training group and the other half was classified. Top – the results using our optimal set of weights, Bottom – the results using only the high Fourier parameters.

In order to validate our initial separation success between the various varieties by their morphology, we statistically analyzed the parameters of all groups using Multivariate Analysis of Variance (MANOVA). The results show a distinct separation between all seven groups except for the couple Pinot-Noir and Hadari. Table 1 summarizes the corresponding *P*-values of the significance separation between every pair of grape types. Another validation test that we used is to run a blind test using Discriminant Analysis (DA). In this method, the objects are randomly divided into two sets each consisting of half of the assemblage. Then, the DA analysis used only one half (the training set) to obtain the DA labeling function and used it to label the objects in the other half (the sample set) as a blind test. This analysis was run 1000 times and a summarization of the distribution of these blind tests classification for each grape seed was calculated. Figure 4 shows the results for the DA test when we used our ‘optimal’ set of weights (up) and with only the high Fourier parameter (down). The number of times a pip was classified at any group is displayed as a black-bar. If a pip was always classified to the same group, then a single bar is plotted and it covers the full height of the corresponding group. However, when it was classified to several groups in different trials of the DA, then a corresponding number of bars are displayed, and their heights are proportional to the classifications percentages. The pattern of the black bars demonstrates that most of the pips were recognized properly. Using the ‘optimal’ weights there were only four pips which were not identified correctly in more than 60% of the runs. On the other hand, at the lower figure it is demonstrated that using only the high Fourier coefficients, many pips were mistakenly identified. Nevertheless, both analysis showed very significant separation between the groups, which means that the Fourier coefficients are highly suitable for morphological classification of grape seeds.

**Table 1:** *P*-values for the statistical significance of the 3D morphological imaging data used for the separation between the varieties, using MANOVA.

	Rumi	‘430’	Merlot	Hadari	Pinot-Noir	‘268’	Baluti
Rumi	—	3.4 × 10^−14^	2.7 × 10^−12^	4.4 × 10^−7^	8.6 × 10^−10^	6 × 10^−8^	9.7 × 10^−5^
‘430’	3.4 × 10^−14^	—	2.3 × 10^−11^	3.7 × 10^−11^	5.3 × 10^−12^	3.4 × 10^−14^	5.1 × 10^−13^
Merlot	2.7 × 10^−12^	2.3 × 10^−11^	—	0.0016	6.4 × 10^−4^	1.6 × 10^−11^	8.4 × 10^−11^
Hadari	4.4 × 10^−7^	3.7 × 10^−11^	0.0016	—	0.0593	4.1 × 10^−7^	1.2 × 10^−6^
Pinot-Noir	8.6 × 10^−10^	5.3 × 10^−12^	6.4 × 10^−4^	0.0593	—	9.9 × 10^−10^	7 × 10^−7^
‘268’	6 × 10^−8^	3.4 × 10^−14^	1.6 × 10^−11^	4.1 × 10^−7^	9.9 × 10^−10^	—	1.4 × 10^−10^
Baluti	9.7 × 10^−5^	5.1 × 10^−13^	8.4 × 10^−11^	1.2 × 10^−6^	7 × 10^−7^	1.4 × 10^−10^	—

**Figure 5:**
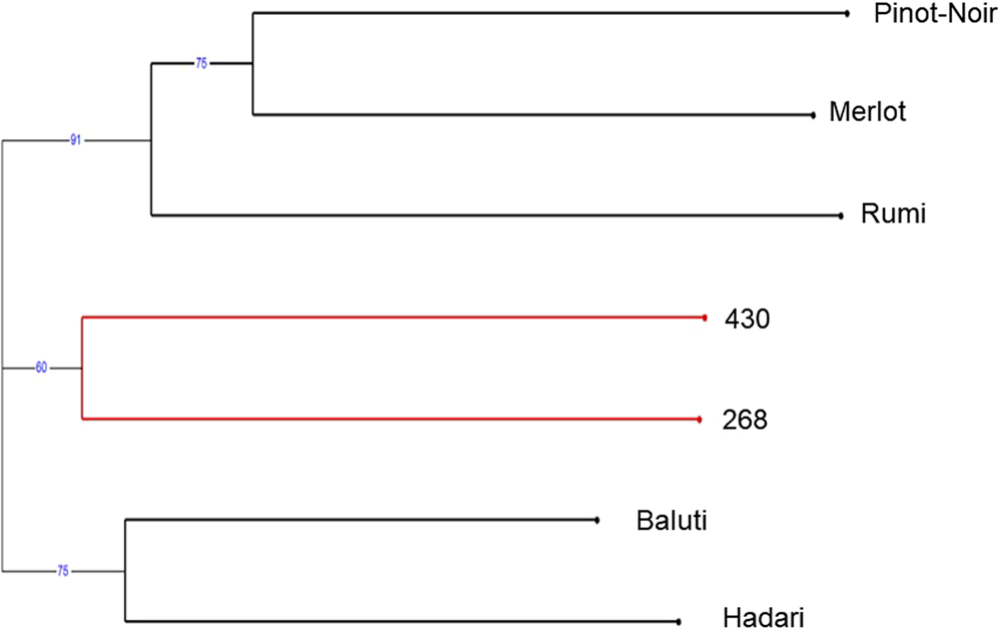
Neighbor-joining dendrogram based on simple matching dissimilarity matrix calculated from the genetic analysis dataset of 22 SSR across 7 genotypes. Red line - *Vitis vinifera* ssp*. sylvestris*. Black lines - *Vitis vinifera* ssp*. sativa*.

In continuation to the success in separating the varieties by their morphological traits, we compared these results with those of SSR analysis of genetic relatedness between varieties. A Neighbor-joining dendrogram based on simple matching dissimilarity matrix (Fig. 5) was calculated from the dataset of 22 SSR across the seven genotypes, which were reported previously^16^. This dendrogram shows a slightly different picture: The Merlot, Pinot-Noir and Rumi varieties cluster together. A second cluster contained Baluti and Hadari varieties, while the *sylvestris* accessions ‘430’ and ‘268’ cluster separately.

Following the initial success in separation of varieties by their 3D structure, we wished to use this tool to analyze the morphological resemblance between couples of varieties, which are named differently by traditional farmers, but are assumed by us to be synonyms. These couples were marked as synonyms in our collection due to the fact that their 22 SSR profiles are identical^16^. The SSR identities are presented in matching colors in Supplementary Information Table S1. Nevertheless, these varieties were preserved for centuries by local farmers, maintaining their names, and we cannot discard the possibility that indeed they are different. This could be explained due to single mutations or other genetic phenomena, which can confer phenotypic differences between those varieties, even though their SSR profiles are identical. We hypothesized that such morphological differences could become apparent in the seed structure amongst other morphological criteria.

**Figure 6:**
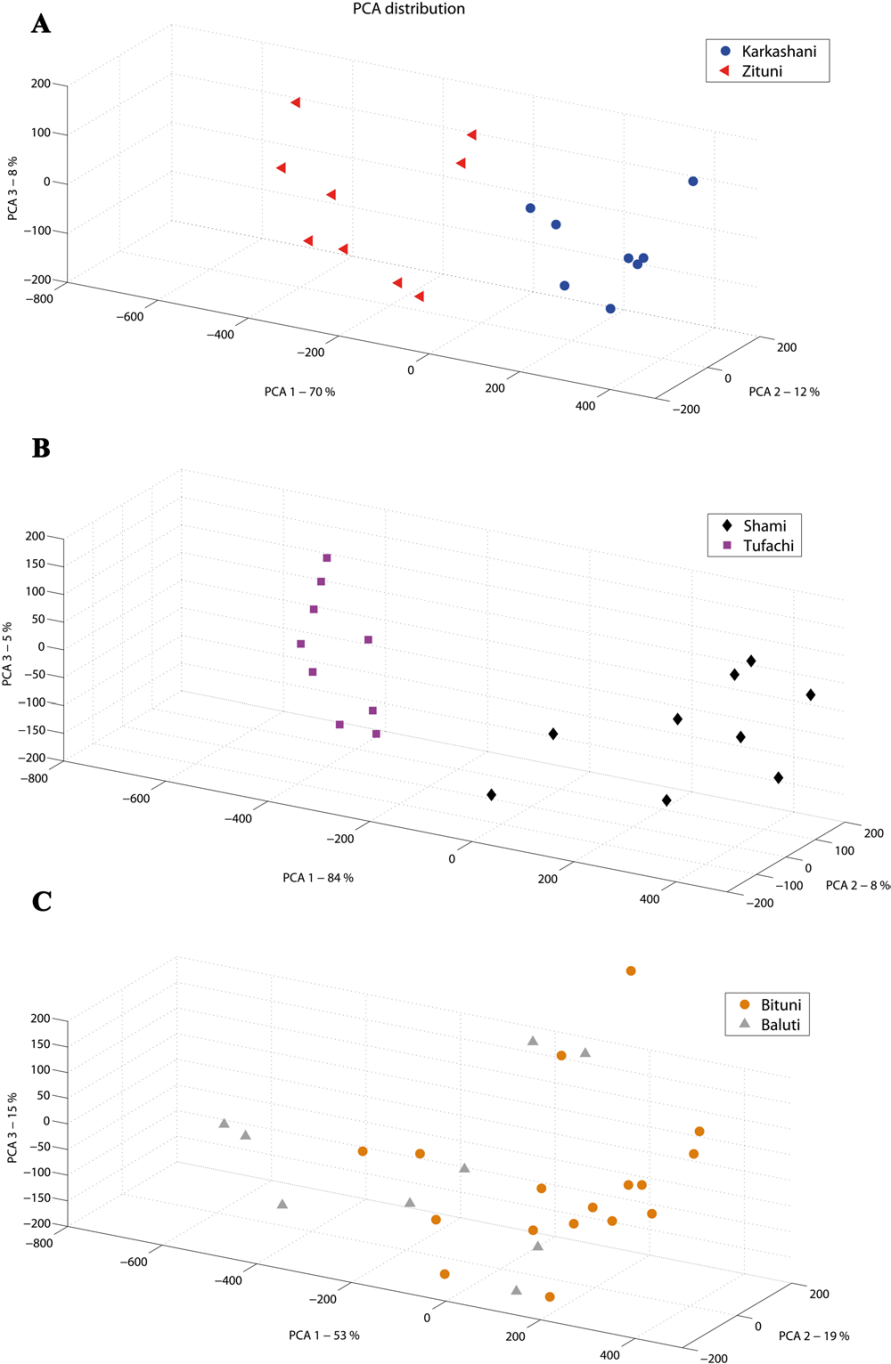
PCA distribution of the 3D morphological values assemblage for 3 couples of varieties, found to have identical 22 SSR genetic fingerprint, as shown in Supplementary Information Table S1. a - Shami/Tufahi. b - Karkashani/Zituni. c - Bituni/Baluti.

We applied the above-mentioned method to compare the assemblage of each of these couples. Two couples, Karkashani/Zituni and Shami/Tufachi (Fig. 6A and B, respectively), showed a clear separation of the seed groups with *P* < 0.000 using MANOVA test, while the couple Bituni/Baluti is less distinctive with *P* = 0.02 for the significance of their separation (Fig. 6C).

## Discussion

We show here for the first time the development of an innovative method aimed for the morphological discrimination between grape varieties, using high resolution 3D data. We show that this method enables us to separate with high statistical certainty between different *Vitis vinifera* varieties. Although the amounts of scanned seeds per each type are not large (e.g. 10–16) the groups are very well separated. The *Vitis vinifera* ssp*. sylvestris* accessions ‘430’ and ‘268’ are well segregated from the rest, both on the PCA plot and on the cluster tree. Four of the *sativa* varieties, namely Rumi, Baluti, Merlot and Pinot-Noir are relatively well separated, with minor overlaps. For instance, two seeds of the Baluti variety are clustered together with Rumi. Still, the clouds of points at the PCA plot and the branches in the cluster tree correspond very nicely to the bulk of six out of seven groups. MANOVA test that was conducted on their PCA distribution proved that these six groups are significantly different from each other. On the other hand, the variety Hadari was not well separated from other varieties in the cluster tree. This is possibly due to the fact that this variety tends to have extremely non-unite berry sizes, possibly making their seeds more polymorphic (see Supplementary Fig. S2). Nevertheless, when using the DA blind test analysis, good identification results were achieved for all varieties, including Hadari.

The morphological analysis was based on Fourier coefficients of several cross-sections that were treated as planar curves. These coefficients were integrated in our analysis together with a set of weights that determines the influence of each parameter. We have tested many sets of weights and selected the one which shows the best separation between the varieties. It is important to note that small changes of the weights didn’t change much the results. Moreover, changing the weights as we wish, does not affect the objectivity of the analysis. This is because every set of weights is based only on intrinsic parameters of the morphology of the seeds, and the classification is repeatable by anyone that will use the same weights. One can think of it as measuring different features like width, height, thickness etc. and then realizing that some of these features are similar over the complete assemblage with no significant variability between groups. In that case, it is completely objective to ignore the irrelevant parameters and base the classification only on the relevant ones.

Indeed, there are less optimal sets of weights for which the separation between the types would not be so clear, due to smaller variability of those weight between varieties. As an example, we show in Supplementary Information Fig. S1 the results with less optimal set of weights. The larger overlap between the types is evident and it means that there are some shape parameters which cannot be used to distinct between varieties of pips. Correlatedly, we show in Supplementary Information Table S2 the corresponding *P*-values of the MANOVA analysis. Many of the results here show considerable overlap between varieties. In our future research, we plan to apply algorithms from the field of machine-learning and artificial intelligence to reveal the optimal set of weights for optimal variety separation. This field of study was developed dramatically during the last two decades and we believe that it has an exciting potential for the question of morphological classification, especially if it is based on significant parameters such as the Fourier coefficients, as we have shown in the current paper.

The SSR analysis of the seven morphologically analyzed varieties showed a clustering profile different from the one obtained by our 3D analysis. In the neighbor joining analysis the European *Vitis vinifera* ssp. *sativa* varieties Pinot-Noir and Merlot were clustered together with the Israeli local variety Rumi, the other two Israeli local varieties Baluti and Hadari clustered together, and the two *sylvestris* accessions clustered separately. This separation is in accordance with our previous results^16^ showing that some Israeli local varieties are genetically closer to European varieties, due to possible late introduction of European plant material to Israel. In addition, the *sylvestris* accessions are generally genetically separated from the *sativa* groups. Nevertheless, this clustering profile is not identical to the morphological one, which means that the two methods are independent and that morphological characterization doesn’t necessarily correspond to the results of SSR relatedness analysis of varieties.

Finally, we morphologically analyzed synonym couples, shown previously to be identical by 22 SSR profile. The SSR analysis by 22 markers is considered today the gold standard method of separating between non-identical varieties of grapevines^19^. Our morphological analysis of grape seeds from these varieties showed that for two couples: Karkashani/Zituni and Shami/Tufachi, a clear separation into two distinct groups was observed, while we found no clear separation of the Bituni/Baluti couple. We propose here that though the couples Karkashani/Zituni and Shami/Tufachi are considered to be synonyms by SSR genetic analysis, they are actually very closely related but not identical, while the assumption of the Bituni/Baluti couple being synonyms was strengthened by the analysis. These results reopen the discussion of the definition of varieties, and the ability to genetically separate between them by SSR analysis. It is well known and was previously demonstrated that SNPs and indels can dramatically change important traits such as berry color^21,22^ and disease resistance^23,24^, and it is indeed logic that other morphological or physiological traits could be altered by specific changes undetected by SSR analysis, thus making two varieties which have identical SSR profiles and are considered synonyms, in fact different in appearance or performance. We believe that future development and increased availability of Next Generation Sequencing tools would enable in the near future the adoption of more accurate tool for genetic analysis, setting a new standard for the genetic characterization of varieties. In the meantime, to our opinion, an effort should be made to preserve national germplasm, including synonyms, which could prove a genetic treasure in the future.

## Conclusions

In this paper, it was clearly demonstrated that the 3D method described is a promising tool for grape variety identification as a case study. It enables us to separate between different *Vitis vinifera* varieties with high statistical certainty. Its novelty lies in the combination of state of the art 3D scanning methods, and the right selection of Fourier coefficients and their weights. In comparing our newly developed method to that of the gold standard SSR analysis, we show that our method can detect morphological differences between previously considered “synonym” couples. This demonstrates the importance of the morphological tool, and presents us with the future prospect of the combination of both methods for higher resolution of variety identification, in *V. vinifera* and other species.

## Material and Methods

### Plant material

Grapes were collected from international varieties growing in commercial vineyards at the region of Shilo, or from the endogenous varieties vineyard collections in Ariel, Sataf and Neve-Yaar, Israel. Mature seeds were extracted from ripened grapes, collected from at least three different grapevines. The seeds were washed by water to discard any residual pulp tissue and air dried for two days, then stored in a closed vial until used. Before 3D scan, each seed was carefully cleaned by brushes and needles from any external tissues which were coating the crevices in the seed in order to enable the effective scan of the seeds’ topography. 10–16 seeds were prepared from each variety for the scans.

### 3D data acquisition

We scanned each seed using a high resolution 3D scanner - ‘PT-M’ (produced by Polymetric GmbH, Darmstadt, Germany), which is based on structured-light technology. The scanner is equipped with two cameras and a projector, which project a unique light pattern on the object. The differences of the returned light between the cameras are the basis for the measurement of the surface. We used the scanner with 75 mm lenses from a distance of 43 cm. The resulted RMS accuracy of the depth measurement was 10 micron and the averaged density of points on the surface is about 25 microns. Two examples of scanned seeds with five perpendicular views and cross-sections can be seen in Fig. 1. Those views were rendered after an automatic positioning procedure as will be explained below.

### Automatic positioning

A crucial step before any comparison of shapes is to have a robust and systematic method of positioning the object that enables precise and repeatable measurements. Only such positioning enables comparing one scan to the other. We used the eigenvectors of the inertia tensor, which is a well-known concept in classical mechanics. It determines the way by which an asymmetric body responds to an applied torque. The eigenvectors of the inertia tensor ordered by the magnitude of the corresponding eigenvalues, set three orthogonal principal axes such that torques around these axes act independently of each other^25^. The first axis is the one around which the body can rotate with minimal force. The second axis is, again, the one around which the body can rotate with minimal force but is perpendicular to the first axis. Similarly, the third axis is defined in the same way and must be perpendicular to the first two axes. This method was used to position flint objects in several studies and proved to be relevant and coherent^11^. Its main advantages are that it is objective, repeatable, based on intrinsic parameters and takes into account the entire shape. The views in Fig. 1 correspond to the directions of the three principal axes of the inertia tensor. The origin of the coordinate system is set to be at the center of gravity of the object. The first axis cuts through the tip of the seeds which is demonstrated in views c,d and e by the fact that the tip is straight upwards. The second and third axes follow the main features of the seeds and its symmetry between right and left, as can be seen in views a,f and g.

### Shape characteristics

Having an automatic and objective method that positions all seeds in the exact same way enabled us to extract a concise, though significant, shape characteristics for the comparisons of the seeds. We have decided to characterize the 3D information using several planar curves which represent, in a very summarizing way, key features of the 3D object. Two representative planar curves are shown in Fig. 1 views 1-f and 2-f. Another significant planar curve that we used is the silhouette of the seeds extracted from their optimal positioning, as can be seen in view b of each example.

By representing these sections as planar curves given with the radius as a function of the arc-length, it is customary to use the Fourier coefficients to define a set of deformation levels which will be used for their descriptions and comparisons. The deformation parameters ${r}_{0},{r}_{1},{r}_{2},\ldots ,{r}_{n}$ represent assorted levels of shape magnitude. The parameter ${r}_{0}$ is the mean radius, and it serves to set the scale of the section. ${r}_{1}$ represents the distance of the curve from the origin of the coordinates system, and therefore the section is determined in an unambiguous way when we choose the origin of the curve such that the coefficient ${r}_{1}$ vanishes. For simple shapes, this choice is equivalent to setting the origin at the center of gravity of the curve at the point (0,0). The first non-trivial coefficient is ${r}_{2}$. It determines the parameters of the ellipse which fits the curve best. ${r}_{2}/{r}_{0}\,\,$is proportional to the eccentricity of that ellipse. The higher order parameters provide information on deformations on smaller scales. However, at some level these parameters represent minute deformations in pips structure that are irrelevant for the identification of the average shape of each variety.

**Figure 7:**
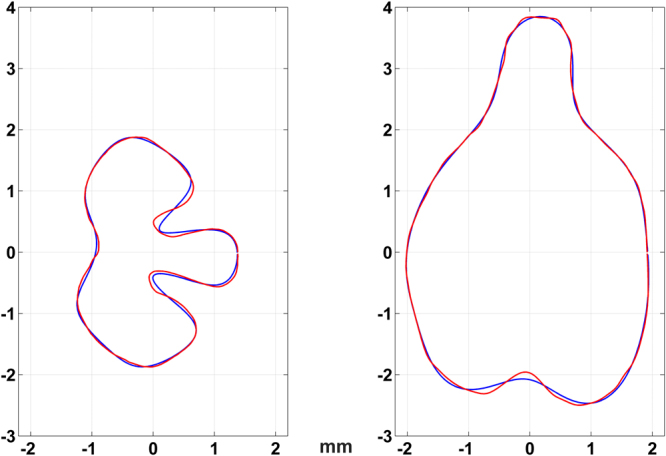
A comparison for one horizontal section (left) and one silhouette (right) between the original cross-sections in red and the curves which represent only the relevant Fourier parameters.

Since deformations, even very small ones, may add noise to the analysis, we used only the size of the section as represented by ${r}_{0}$ together with the first six non-trivial parameters $\,{r}_{2}-{r}_{7}$, in the analysis. Figure 7 shows how the sections would look like if we ignore the rest of the Fourier parameters $\,{r}_{8},{r}_{9},\ldots$ , and up. The Red lines represent the original curves and the blue lines are the same curves after the high Fourier coefficients were ignored. The two lines are very similar but there are some differences that were smoothed to avoid the noisy data.

We summarize this section by noting that for each 3D model we have computed five horizontal sections in equidistant steps and one silhouette. We represent each of these curves using seven parameters, meaning that the complete 3D morphological data is represented now with a row vector of 42 values $\,[{v}_{1},{v}_{2},\ldots ,{v}_{42}]$. The first 35 parameters correspond to the five horizontal sections (seven values per one section), and the last seven parameters correspond to the Fourier coefficients of the vertical silhouette.

### Distance function

The final automatic classification of the seeds is based on well-known statistical methods such as Cluster Analysis (CA) and PCA. These methods make use of a distance matrix from which the grouping can be observed.

We have defined the distance between any pair of seeds $(\alpha ,\beta )$ in terms of a weighted Euclidian distance between their corresponding vectors of Fourier parameters:

1 $$d(\alpha ,\beta )=\sqrt{{\sum }_{i=1}^{42}{\omega }_{i}\cdot {({v}_{{\alpha }_{i}}-{v}_{{\beta }_{i}})}^{2}}.$$

While the weights ${\omega }_{i}$ are set to satisfy ${\omega }_{1}+{\omega }_{2}+\ldots +{\omega }_{42}=1$, and they give us the freedom to emphasize some of the parameters more than others. For instance, to give more weight to the size $({r}_{0})$ of the seed or to highlight the elliptic coefficient $({r}_{2})\,\,$instead of higher order coefficients $({r}_{3}-{r}_{7})$. The exact set of weights that we used are specified below.

### Automatic Classification

As was mentioned above, once the distance matrices are given there are many combinations and classification methods that can be used. Here, we chose an iterative procedure that is based on CA and PCA, as it was proved successful for automatic classification of ceramic shapes^26^. There are several steps in the procedure and in each of them the subgroups which were defined in the former phase are reclassified based on a new set of weights. For instance, CA with high weights to the size of the seeds would split the assemblage into several subgroups on the main branches of the cluster tree (three in our case, see Fig. 3). But, in the next step, when on every branch there are already seeds of similar sizes, it is more reasonable to further classify them based on different parameters, such as the eccentricity of the sections $({r}_{2})$ or higher Fourier coefficients. At the final results we did three iterations and we used the following set of weights: for the initial classification we gave total of 25% weight to the size of the sections parameters $[{v}_{1},{v}_{8},{v}_{15},{v}_{22},{v}_{29},{v}_{36}-{r}_{0}]$. Another 25% were given to the elliptic coefficient (parameters $[{v}_{2},{v}_{9},{v}_{16},{v}_{23},{v}_{30},{v}_{37}-{r}_{2}]$), and 50% of the weight was given to the rest of the parameters equally. At the second and third iterations, we reduced the weight of ${r}_{0}$ to only 15% and added it to the higher Fourier coefficients with a total of 60%, leaving the ${r}_{2}$ parameters with 25%.

Obviously, there is unlimited number of options to run the analysis, where in every step one can change the weights. However, many of them are redundant and reveal very similar results. Our conclusions and results are based on the global picture which came out of many of the weighted combinations, which showed very similar classifications and separated between the different types. We refer to this set of weights as ‘optimal’ and use them in the presented analysis. Moreover, to assert the statistical significance of the classification offered by the PCA and the CA, we used Discriminant Analysis (DA). In this method, the objects are randomly divided into two sets each consisting of half of the assemblage. Then, the DA analysis is using only one half (the training set) to obtain the DA labeling function, and use it to label the objects in the other half (the sample set) as a blind test. Thus, every object in the sample set is classified into one of the groups based on the DA function of the training set. Repeating this process many times each object appears approximately half of the times in the sample set, and acquires a corresponding number of labels. If the resulting labels distribute narrowly at a distinct group, it means that the original grouping are well separated, and the classification considered significant.

### Genetic analysis by SSR

In order to determine the genetic relations between the various varieties presented in this work, we used the standard 22 SSR analysis approach^19^, of which data we collected previously from a large set of local varieties^16^. SSRs were analyzed by the same methods for the international varieties presented in this work. An unweighted neighbor-joining tree was constructed using the sizes of the 22 SSRs for each variety, based on the simple-matching dissimilarity matrix with 10,000 bootstrap replicates. This analysis was performed using the Darwin software package v6^27^. Following the analysis, the neighbor joining tree demonstrates the relatedness or distance between varieties, which are presented close or far from each other on the tree, relatively.

### Data availability statement

The datasets analyzed during the current study were previously published by Drori *et al*. (2017), and are available online.

## Electronic supplementary material

supplementary information
