# Supplementary material for: Development of a 3D seed morphological tool for grapevine variety identification, and its comparison with SSR analysis
Source: Sci Rep. 2018 Apr 25;8:6545. doi: 10.1038/s41598-018-24738-9 (PMC5917028; doi:10.1038/s41598-018-24738-9)
Supplement: Supplementary file 1 — supplementary information [file 41598_2018_24738_MOESM1_ESM.docx]

**Development of a 3D seed morphological tool for grapevine variety identification, and its comparison with SSR analysis**

Avshalom Karasik^1^, Oshrit Rahimi^2^, Michal David^3^, Ehud Weiss^3^ * and Elyashiv Drori^4, 2^ *

Supplementary Information - legends

**Fig. S1** PCA distribution of the assemblage after giving all of the weight to the high Fourier coefficients r_4-r_7. The mixture between the seven types is prominent relatively to Fig. 2, and well-explain why we do not recommend them.

**Fig. S2** Hadari grape bunch – this local grape variety has large polymorphism in berry size, resulting in different seed sizes and possibly – structure. Picture at the curtesy of Yonatan Kanaskevich, Israel.

**Table S1** SSR analysis of 12 grapevine varieties analyzed in this work. The analysis was done using the standard 22 SSR marker sizes acquired by PCR amplification by specific primers (i.e. VVMD27, VVS2, etc.). Varieties marked by the same color were found to be genetically identical by the SSR analysis.

**Table S2** P-values for the statistical significance of the separation between the grapevine varieties using MANOVA, following the use of a biased set of weights (high Fourier coefficients r_4-r_7.).

**Figures**

**Fig. S1**

**Fig. S2**


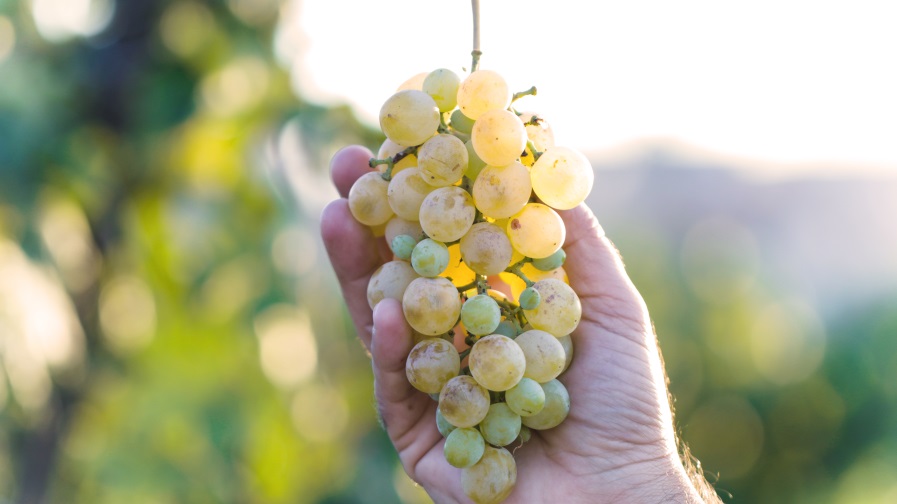


**Tables**

**Table S1**

**Table S2**

|  | Rumi | '430' | Merlot | Hadari | Pinot-Noir | '268' | Baluti |
| --- | --- | --- | --- | --- | --- | --- | --- |
| Rumi | ---- | 0.00 | 0.00 | 0.09 | 0.05 | 0.00 | 0.00 |
| '430' | 0.00 | ---- | 0.00 | 0.00 | 0.00 | 0.02 | 0.34 |
| Merlot | 0.00 | 0.00 | ---- | 0.00 | 0.00 | 0.11 | 0.02 |
| Hadari | 0.09 | 0.00 | 0.00 | ---- | 0.47 | 0.03 | 0.00 |
| Pinot-Noir | 0.05 | 0.00 | 0.00 | 0.47 | ---- | 0.00 | 0.00 |
| '268' | 0.00 | 0.02 | 0.11 | 0.03 | 0.00 | ---- | 0.08 |
| Baluti | 0.00 | 0.34 | 0.02 | 0.00 | 0.00 | 0.08 | ---- |
| Total Group | 0.00 | 0.00 | 0.00 | 0.14 | 0.12 | 0.00 | 0.03 |
